# Macrophage conditioned medium promotes colorectal cancer stem cell phenotype via the hedgehog signaling pathway

**DOI:** 10.1371/journal.pone.0190070

**Published:** 2018-01-02

**Authors:** Fan Fan, Rui Wang, Delphine R. Boulbes, Huiyuan Zhang, Stephanie S. Watowich, Ling Xia, Xiangcang Ye, Rajat Bhattacharya, Lee M. Ellis

**Affiliations:** 1 Department of Surgical Oncology, University of Texas MD Anderson Cancer Center, Holcombe Boulevard, Houston, Texas, United States of America; 2 Department of Immunology, University of Texas MD Anderson Cancer Center, Holcombe Boulevard, Houston, Texas, United States of America; Università degli Studi della Campania "Luigi Vanvitelli", ITALY

## Abstract

**Background:**

There is conflicting data on the role of macrophages in colorectal cancer (CRC); some studies have shown that macrophages can exert an anti-tumor effect whereas others show that macrophages are tumor promoting. We sought to determine the role of conditioned medium (CM) from macrophages, in particular classically activated macrophages, on the development of the CSC phenotype in CRC cells, which is believed to mediate tumor growth and chemoresistance.

**Methods:**

Murine (CT26) and human (HCP-1) CRC cell lines were treated with CM from lipopolysaccharide (LPS)-activated murine macrophages. The CSC population was assessed using the sphere-forming assay and aldehyde dehydrogenase assay. Chemoresistance studies were performed using the MTT assay. CSC transcription factors and SHH protein were analyzed by Western blotting.

**Results:**

The results showed that LPS-activated macrophage CM induced the CSC phenotype in CRC cells. Further studies showed that the CSC phenotype was mediated by the sonic hedgehog (SHH)-Gli signaling pathway, which is known to drive self-renewal; these effects were blocked by depletion of SHH in macrophage CM. In addition, LPS-activated macrophage CM enhanced chemoresistance.

**Conclusions:**

LPS-activated macrophages play an active role in promoting the CSC phenotype through activation of the SHH-Gli signaling pathway in CRC cells.

## Introduction

Colorectal cancer (CRC) is the second leading cause of cancer-related death in the United States [1]. Several clinical trials have led to the approval of new drugs for patients with metastatic CRC (mCRC) within the past few years; however, the prognosis remains poor, and most patients will develop resistance to therapy within one year [1–3]. Thus, finding method to combat resistance to therapy is critical if we are to improve the survival of patients with mCRC.

Cancer stem cells (CSCs), a subpopulation of cancer cells that are thought to drive tumor growth and metastasis, have been identified in multiple types of malignancies, including CRC [4–13]. CSCs are thought to not only initiate and sustain tumor growth but also mediate chemoresistance [14–20], mostly through key signaling pathways such as Wnt, Notch, and Hedgehog [21]. Notably, results from our laboratory and others suggest that CSCs exist in a state of flux and that the CSC phenotype can be enhanced by micro-environmental influences [22–26]. Currently most therapies targeting the bulk of the tumor cells ultimately fail as they do not successfully eliminate CSCs. Therefore, understanding the CSC-microenvironment relationship and the development of CSC- or microenvironment-targeting strategies that could eliminate or deplete the CSC population are critical for improving the clinical outcomes of patients with mCRC.

The tumor microenvironment is complex and made up of various cell types, including tumor associated macrophages (TAMs) (reviewed in [27]). Macrophages have been previously shown to interact with stem cells [28], and a growing body of evidence suggests that TAMs are critical for the self-renewal and maintenance of CSCs in established tumors [29–31]. Recent studies have sought to characterize the relationship and cross-talk between TAMs and CSCs (reviewed in [30]) and have demonstrated that TAMs are able to increase the number of CSCs and cell phenotypes in hepatocellular carcinoma and pancreatic ductal carcinoma [32–34]. Also, it was shown that TAMs can increase both tumorigenicity and drug resistance in CRC both *in vitro* and *in vivo* [35]. These effects were due to coordinated activation of Stat3 and Sonic Hedgehog (SHH) signaling in CRC stem cells. However, despite these exciting results, there is still a tremendous amount to learn about the interactions between TAMs and CSCs, particularly in CRC.

Typically, macrophages are divided into two main classes, M1 (classically activated macrophages; antitumor activities) and M2 (alternatively activated-macrophages: protumor activities) [29, 36]. However, the role of different macrophages in the CRC microenvironment are not well defined and remains a point of controversy (reviewed in [37–39]). In vitro, exposure to lipopolysaccharide (LPS) polarizes macrophages toward an M1 phenotype, whereas exposure to IL-4 or IL-13 polarizes them towards an M2 phenotype. In this study, we examined the effects of classically LPS-activated macrophage CM on the acquisition of the CSC phenotype in CRC cells. Our studies demonstrated that the CM of LPS-activated macrophages is able to increase the CSC phenotype and promote chemoresistance of CRC cells. We also showed that the CSC phenotype was enhanced by the secretion of sonic hedgehog (SHH) by LPS-activated macrophages.

## Materials and methods

### Cell lines

Freshly isolated HCP-1 CRC cells were established in our laboratory, as previously described [40]. The murine cell lines CT26 and RAW264.7 (hereafter RAW) and the human monocyte cell line U937 were purchased from American Type Culture Collection (Manassas, VA, USA). CT26 and RAW cells were maintained in culture using standard protocols in minimal essential medium, supplemented with 10% fetal bovine serum (FBS) at 37°C in 5% CO_2_. U937 cells were maintained in RPMI 1640 medium supplemented with 10% FBS, 2 mmol/L L-glutamine, and 0.05 mM 2-mercaptoethanol. Cells were confirmed to be free of mycoplasma using the MycoAlert mycoplasma detection kit (Lonza Group, Allendale, NJ). The results of all *in vitro* studies were reproduced in at least three independent experiments.

### Macrophage differentiation

Human blood was obtained from healthy (anonymous) donors at the Gulf Coast Regional Blood Center, Houston TX, and was purchased the Blood Center with an IRB exemption. The monocytes were obtained from buffy coat by gradient centrifugation using Ficoll-Paque (GE Healthcare Life Sciences). Non-adherent cells were removed and purified monocytes were incubated for 7 days in RPMI 1640 supplemented with 10% FBS and 50 ng/ml M-CSF to obtain macrophages (hereafter Human Primary Macrophages). Cells were washed with PBS twice and incubated overnight with 10% FBS-MEM supplemented with 1 μg/ml of LPS (Sigma, St. Louis, MO, USA). Cells were then washed with PBS twice and cultured with MEM-1% FBS for 48 h. The conditioned medium was harvested and filtered through a 0.22-μm filter to remove cell debris before being added to the CRC cell cultures.

### Conditioned medium preparation

CT26, HCP-1, and RAW cells were cultured under MEM-1% FBS conditions for 48 h. The media were harvested and filtered through a 0.22-μm filter to remove cell debris and serve as a control. Murine RAW macrophages and human U937 monocytes were activated using 1 μg/ml of LPS solution and incubation overnight. Cells were then washed with PBS twice and cultured with MEM-1% FBS for 48 h. The media were harvested and filtered through a 0.22-μm filter to remove cell debris before being added to the CRC cell cultures.

### MTT assay

Pretreated CRC cells with CM for 48 h, cells were then trypsinized and seeded 3,000 cells/well with CM with or without 5FU or SN38 into the 96 well plates and the cells were incubated for 72 h. At the end of the incubation, 3- [4, 5-dimethyl-thiazol-2-yl] 2, 5 diphenyltetrazolium bromide (MTT; Sigma) was added to a final concentration of 0.5 mg/ml, and the cells were incubated for another 2 h. After the medium and MTT were removed, dimethyl sulfoxide was added for 1 min, and absorption was read at 570 nm.

### Aldefluor assay

The Aldefluor kit from Stemcell Technologies (Vancouver, CA) was used to identify cells that exhibited high ALDH enzymatic activity, according to the manufacturer’s instructions. In brief, cells were trypsinized and suspended in Aldefluor assay buffer containing ALDH substrate (BAAA, 1 μmol/L) and incubated at 37°C for 30 minutes. As a negative control, an aliquot from each sample was treated with 50 mmol/L diethyl-aminobenzaldehyde, a specific ALDH inhibitor, and followed up by flow cytometric analysis using FlowJo software (Tree Star, Inc., Ashland, OR).

### Sphere-forming assay

CT26 and HCP-1 cells were plated in 96-well, ultra-low-attachment plates (BD Biosciences, San Jose, CA) at a density of 50 or 100 viable cells per well, respectively. Standard sphere-forming medium (serum-free DMEM/F-12 supplemented with 1× B27 serum substitute, 20 ng/ml human recombinant epidermal growth factor, and 20 ng/ml basic fibroblast growth factor [all from Invitrogen, Carlsbad, CA]) was mixed at a 1:3 ratio with macrophage CM or control CM and added to the CRC cells. Plates were incubated at 37°C and 5% CO_2_ and cultured for 7–14 days. Spheres larger than 50 μm in diameter were counted. For siRNA knockdown, cells were transfected with siRNAs, recovered overnight in normal growth medium, and then the cells were single suspended and seeded 100 cells /well as described above. For assays with the smoothened (SMO) inhibitor, LDE225 (Selleckchem Houston, TX), cells were pretreated with the inhibitor at indicated doses for 4 hours, and then the cells were single suspended and seeded at 100 cells / well as described above with inhibitor.

### Promoter reporter assay

HCP-1 cells were infected with lentiviruses containing a Hes-1 AB Notch/CSL luciferase reporter [41], a pWPT-Gli-GFP reporter, or a pWPT-TCF-GFP reporter (kind gifts from Dr. Renuka P. Limgala, NIH [42]). The reporter-containing CRC cells were then incubated with LPS-activated macrophage CM. After 24 h of culture, promoter activities were determined by FACS analysis.

### Western blot analysis

Cell lysates or concentrated CM (concentrated by P3 Ultra-4 centrifugal filter units [Millipore]) were run on SDS-PAGE, following a standard protocol. The following primary antibodies were used: Gli, OCT/4, β-Catenin (Cell Signaling, Danvers, MA), Hes-1 (Abcam, Cambridge, MA), rabbit polyclonal antibody SHH, Nanog, and β-actin (Santa Cruz, Dallas, TX). Signals were detected by chemoluminescence (Fisher Scientific, Pittsburgh, PA).

### Reverse transcription polymerase chain reaction

RNA from RAW or RAW+LPS cells was isolated by TRIzol extraction (Invitrogen) and purified using the RNeasy kit (Qiagen, Valencia, CA). First-strand cDNA was synthesized with SuperScript III reverse transcriptase (Invitrogen). PCR amplification for M1 or M2 macrophage markers was performed under the following conditions: 95°C for 5 min, 25 cycles of 30 s denaturation at 95°C, 30 s annealing at 60°C, and 1 minute of extension at 72°C. Products were analyzed by electrophoresis of 20 μL of each PCR reaction mixture in a 1.5% agarose gel, and bands were visualized by ethidium bromide staining. The primers used are presented in S1 Table.

### siRNA knockdown

A mixture of two different small interfering RNAs (siRNAs) targeting SHH (designed by multiple siRNA design platforms) was used to deplete SHH in RAW cells. The sequences were #1 sense 5’ CAUCCACUGUUCUGUGAAA, antisense 5’ UUUCACAGAACAGUGGAUG; and #2 sense 5’ GGGUCUACUAUGAAUCCAA, antisense 5’ UUGGAUUCAUAGUAGACCC. A validated non-specific siRNA (control siRNA) was obtained from Sigma. Lipofectamine 2,000 siRNA transfection reagent was used according to the manufacturer’s instructions (Invitrogen). The siRNA knockdown was performed in Opti-MEM (Gibco). 24 h after transfection, cells were washed with PBS, cultured in MEM-10% FBS supplemented with LPS, and incubated overnight. Cells were then washed with PBS twice and cultured with MEM-1% FBS. 48 h later, supernatant media were collected and assessed for SHH depletion via Western blot analysis.

### Statistical analyses

Statistical analyses were performed using Student’s *t*-test (Microsoft Excel 2007, Redmond, WA). All statistical tests were 2-sided, and *P* values ≤0.05 were considered statistically significant.

## Result

### Murine macrophage CM promotes the CRC stem cell phenotype in murine and human CRC cell lines

The RAW macrophages were first polarized by LPS into classically activated macrophages. The resulting population was characterized by RT-PCR through the detection of M1 and M2 polarization markers, as shown in S1 Table. Our PCR results showed that LPS-activated macrophages, although classically described as M1 macrophages, are actually a mixture of both M1-like and M2-like macrophage phenotypes (S1A Fig).

**Fig 1:**
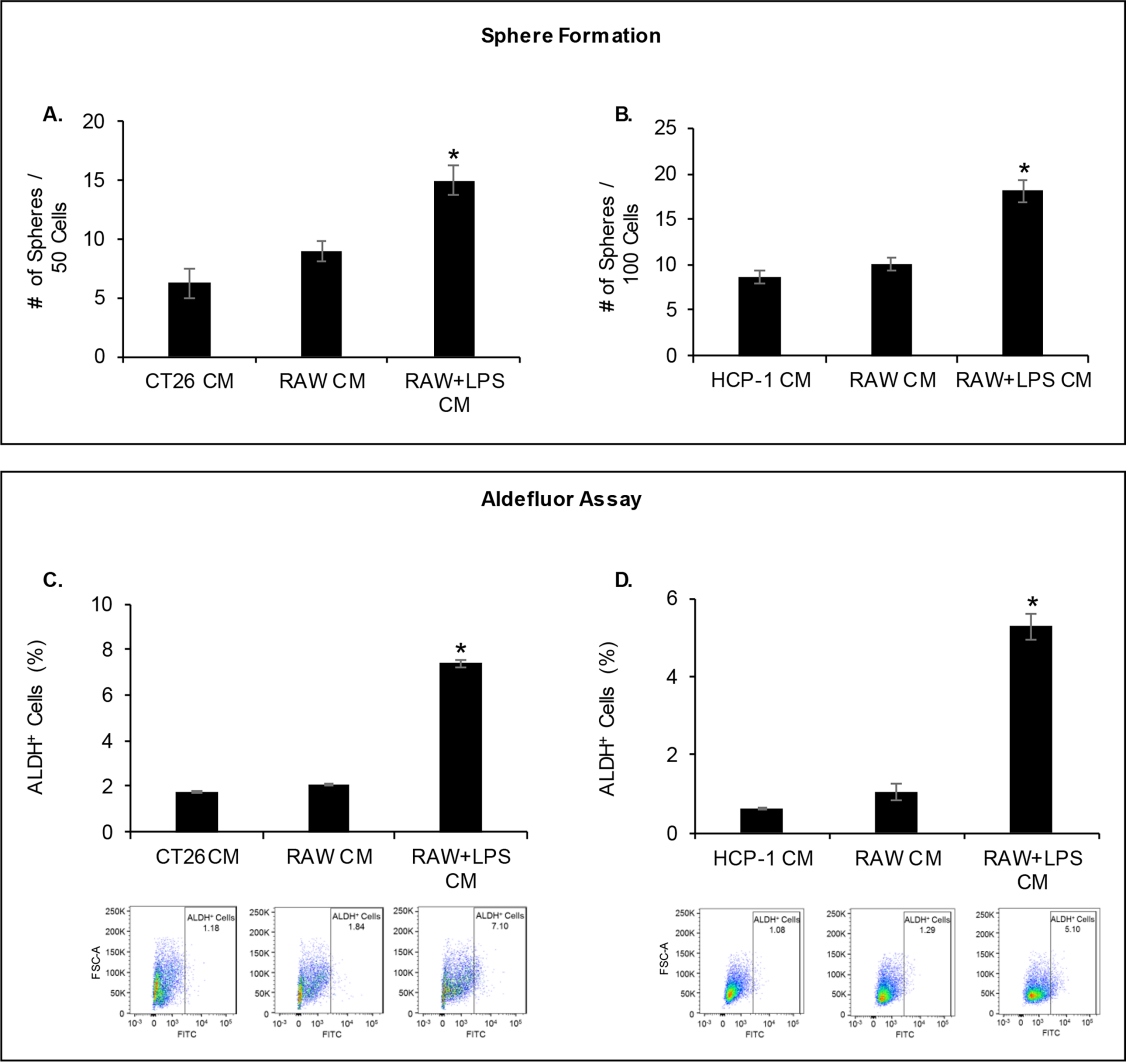
Murine macrophage Conditioned Media (CM) promotes the stem cell phenotype of CRC cells. **(A** and **B)** CT26 and HCP-1 cells were cultured with LPS-activated macrophage CM or control CM, and a sphere-forming assay was performed (*P < 0.01). **(C** and **D)** Aldefluor-positive cell population was determined (*P < 0.001).

To understand the potential role of macrophage-secreted factors in promoting the CSC phenotype of CRC cells, we incubated CT26 and HCP-1 cells with CM obtained from LPS-activated RAW macrophages; in parallel, we performed stimulations with non-activated RAW and CRC cells, using their own CM as the controls. CRC cells were analyzed using two common techniques for detecting the CSC phenotype: sphere-forming and ALDH activity assays. Fig 1A and 1B show that LPS-activated macrophage CM increased the sphere-forming capability of both murine CT26 and human HCP-1 CRC cell lines by more than two-fold compared to the control CM. In addition, LPS-activated macrophage CM treatment enriched the Aldefluor-positive cell population of both CT26 and HCP-1 cells by ~four- and ~eight-fold, respectively, compared to controls (Fig 1C and 1D). As a secondary readout for stem-ness, we also assessed alterations in CD133 positive CRC cells in response to treatment with LPS-activated macrophage CM. Exposure to LPS-activated macrophage CM led to a ~3-fold increase in CD133 positive CRC cells as compared to control or untreated macrophages (data not shown). Together, these data suggest that LPS-activated macrophage-derived soluble factors promote the CSC phenotype of CRC cells in a paracrine manner.

### Murine macrophage CM enhanced the CSC phenotype through the SHH signaling pathway

**Fig 2:**
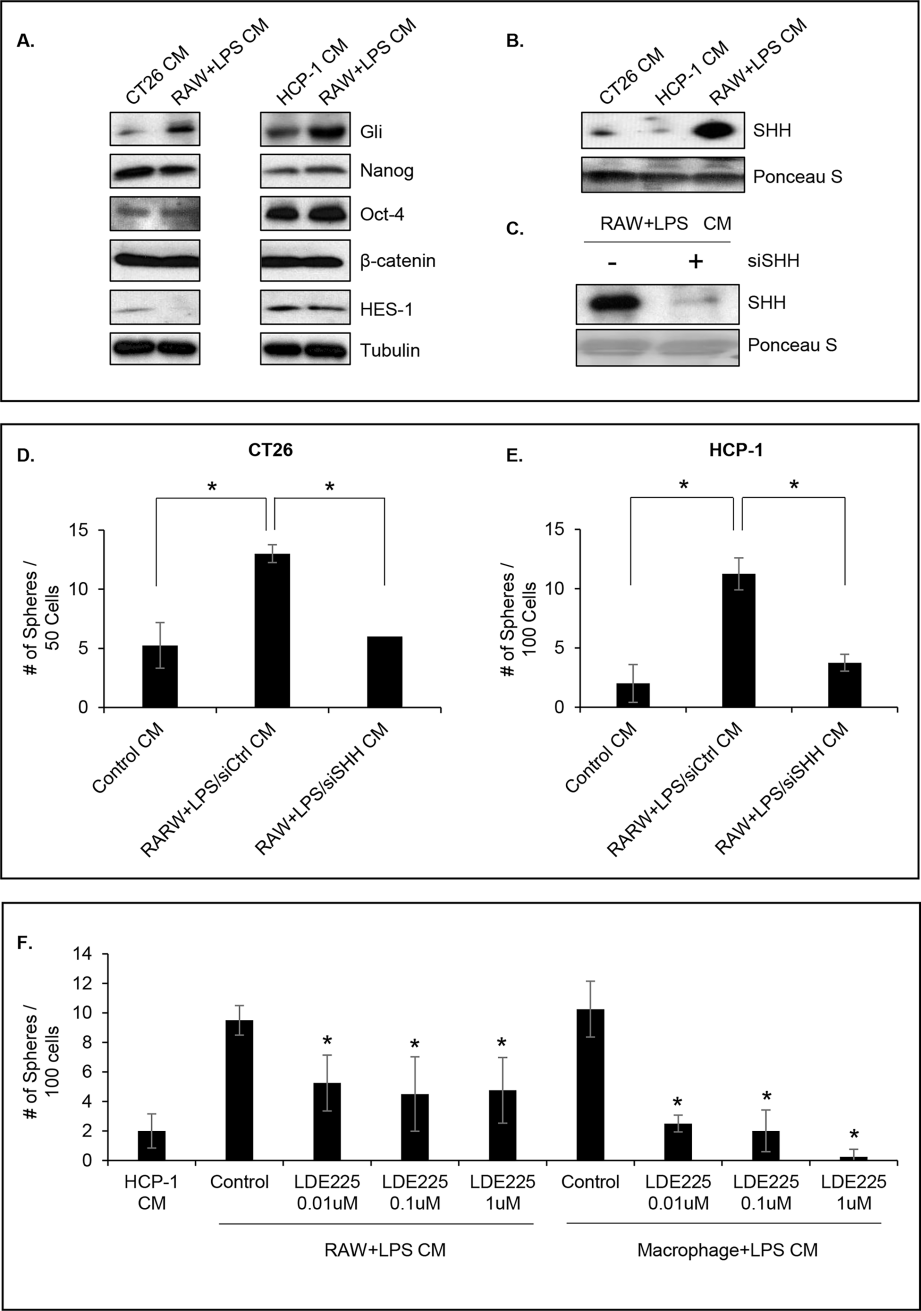
Murine macrophage CM enhanced the SHH signaling pathway. **(A)** CSC transcription factor expression with 48 h of CM treatment in CT26 and HCP-1 cells. **(B)** Western blot analysis of SHH secretion in CRC cells and RAW cells. **(C)** Western blot analysis shows knockdown of SHH at 48 h after the indicated siRNA transfections. **(D** and **E)** Effects of knockdown SHH-mediated sphere formation in CT26 and HCP-1 cells (*P < 0.01). **(F)** HCP-1 cells were cultured with LPS-activated RAW CM or LPS-activated primary macrophage CM with or without a SMO inhibitor and the sphere-forming assay was performed (*P < 0.01).

To understand the underlying mechanism of the activation of the CSC phenotype in CRC cells by LPS-activated macrophage CM, we determined the activity of canonical CSC pathways such as Notch [43], Wnt/β-Catenin [44], and SHH [45] in these cells. To this end, we infected HCP-1 cells with a lentivirus containing a Hes-1, TCF, or Gli promoter-driven reporter and treated them with LPS-activated macrophage CM or control CM for 48 h. LPS-activated macrophage CM treatment led to an increase in the activity of Gli promoter but not of Hes-1 or TCF promoter (S2 Fig). In addition, Western blot analysis of the canonical CSC transcription factors (Gli, HES-1, β-Catenin, Nanog, and OCT-4) confirmed that Gli protein levels were significantly increased following LPS-activated macrophage CM treatment of both CT26 and HCP-1 cells (Fig 2A). Moreover, since the Gli pathway is activated by SHH, we assessed the presence of SHH in CM by Western blot analysis. The data demonstrated that SHH was secreted at high levels by LPS-activated macrophages compared to CT26 and HCP-1 cells (Fig 2B) and suggest that LPS-activated macrophages secrete SHH that, in turn, activates the SHH intracellular pathway in CRC in a paracrine fashion. Of note, there is 92.4% shared identity between human and mouse SHH proteins [46]; therefore, one should not expect inter-species protein interactions to be an issue in the following experiments.

To confirm the hypothesis that the macrophage-secreted SHH is responsible for the increase we previously observed in the CSC population (Fig 1), we used siRNA to knock down SHH expression in RAW cells, thereby decreasing its secretion by macrophages (Fig 2C). The CM were collected from siSHH or control siRNA-transfected macrophages and incubated with CT26 or HCP-1 cells; the CSC population was analyzed using the sphere-forming assay. Fig 2D and 2E show that SHH knockdown dramatically decreased sphere formation in both cell lines (P < 0.01).

Next, we evaluated the effects of a small-molecule inhibitor of SMO, LDE225 ([47]. LDE225 significantly inhibited the sphere forming capacity in HCP-1 cells in a dose dependent manner following RAW + LPS CM and macrophage + LPS CM treatment (Fig 2F, P < 0.01). Taken together, these data confirmed that LPS-activated RAW macrophages, and human macrophage activated by LPS as well secrete SHH, which in turn, increases the CSC population in CRC cells.

### Murine macrophage CM promotes chemoresistance of CRC cells

**Fig 3:**
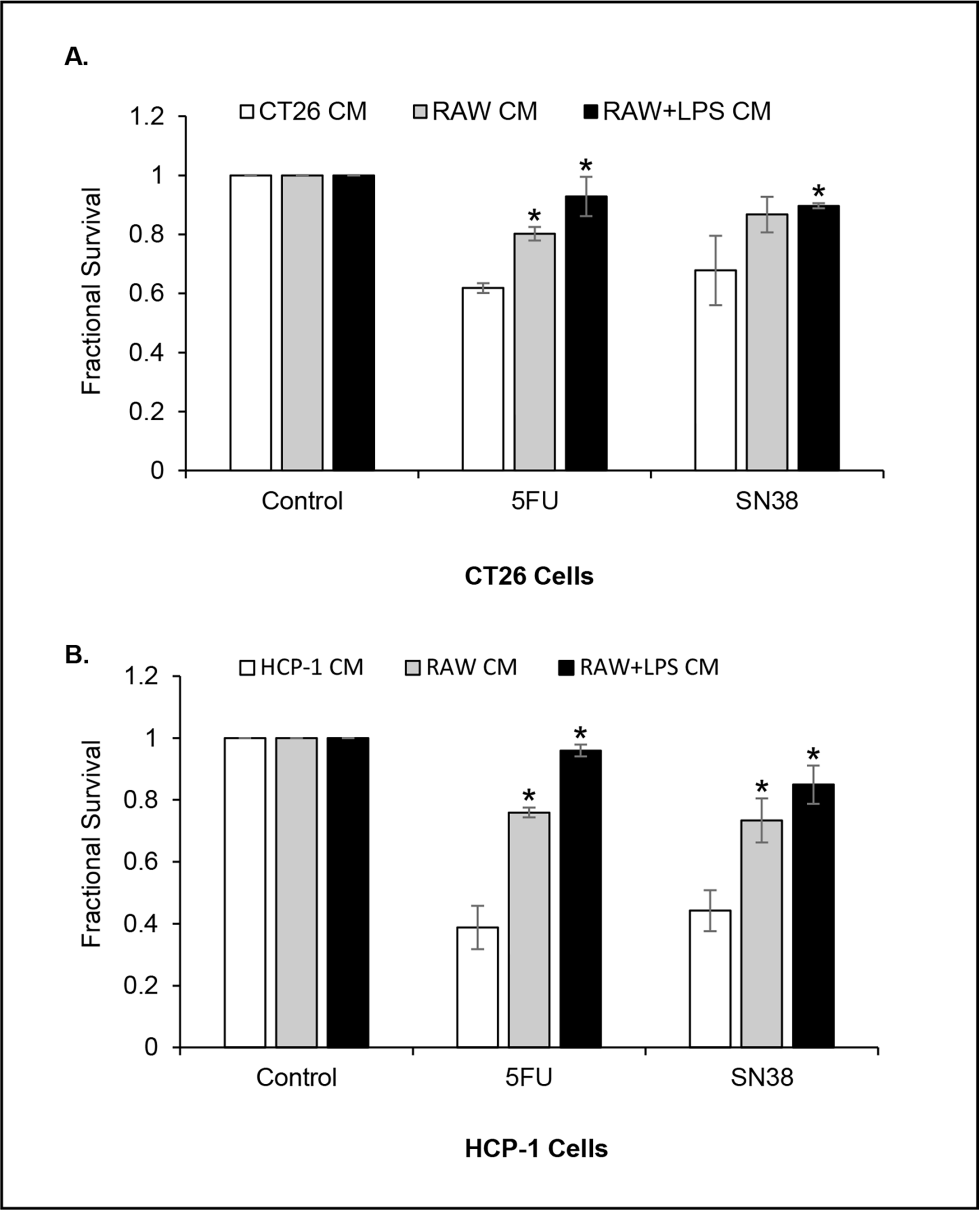
Murine macrophage CM promotes chemoresistance of CRC cells. **(A** and **B)** CM pretreated CT26 or HCP-1 cells were incubated with 5-FU or SN38 in control CM or RAW+LPS CM and cell viability was determined by MTT assay (*P < 0.05; *P < 0.01 respectively).

Preclinical studies suggested that CSCs possess intrinsic properties that mediate their resistance to chemotherapy [16, 17, 48]. Since our previous data showed that macrophage CM enhanced the CSC phenotype, we assessed the CRC cells response to chemotherapy after a 48 h pretreatment with murine macrophage CM. After pretreatment, the CRC cells were exposed to either 5-fluorouracil (5-FU, 2 μg/ml) or SN38 (20 nM) in LPS-activated macrophage CM or control CM for 72 h and cell survival was assessed by the MTT assay. Our results showed that both CRC cell lines grown in CT26 CM or HCP-1 CM exhibited a significantly higher sensitivity to 5FU or SN38, whereas, the cells cultured in LPS-activated macrophage CM demonstrated a significantly higher survival rate after 5-FU or SN38 treatment (Fig 3A and 3B, P < 0.05 vs CT26 CM; P < 0.001 vs HCP-1 CM, respectively). We also observed that non-activated macrophage CM induced resistance to 5FU and SN38 in CRC cells, suggesting that non-activated macrophages can also influence chemoresistance in tumor cells.

### Human macrophage CM promotes the self-renewal capacity of CRC cells

Finally, we confirmed the effect of macrophages CM on CSC using the human U937 monocyte cell line. U937 monocytes were polarized by LPS, and the population was characterized by RT-PCR (S1B Fig). As previously noted with RAW cells, we observed that LPS-activated U937 cells were a mixture of M1-like and M2-like macrophages.

**Fig 4:**
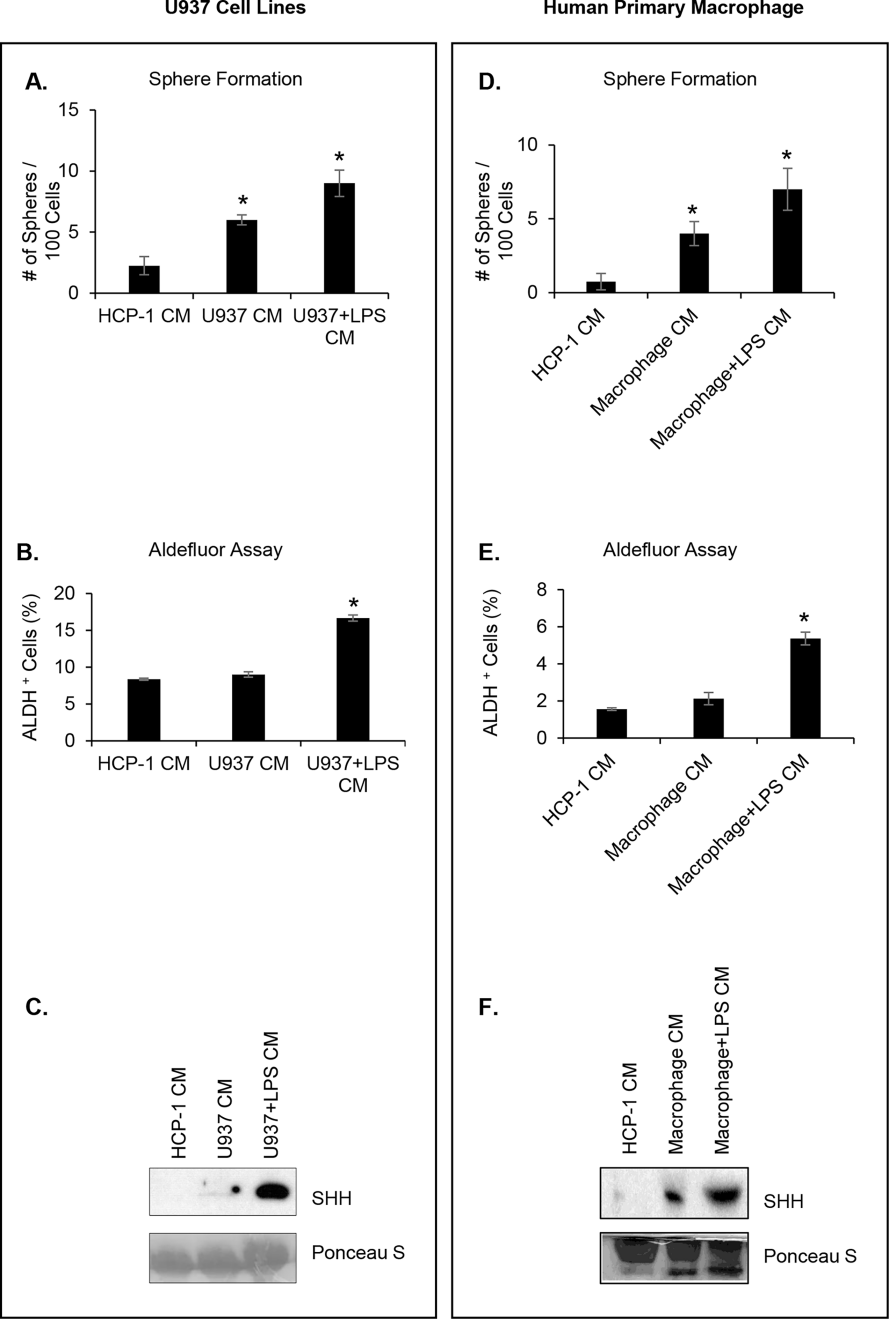
Human macrophage CM promotes the self-renewal capacity of CRC cells. **(A)** HCP-1 cells were cultured with LPS-activated U937 CM or control CM and the sphere forming assay was performed (*P < 0.001). **(B)** The Aldefluor-positive cell population was determined (*P < 0.01). **(C)** Western blot analysis of SHH secretion. Ponceau S served as loading control. **(D)** HCP-1 cells were cultured with LPS-activated human macrophage CM or control CM, sphere forming assay was performed (*P < 0.01). **(E)** The Aldefluor-positive cell population was determined (*P < 0.001). **(F)** Western blot analysis of SHH secretion. Ponceau S served as loading control.

The CM were prepared as previously described. After the HCP-1 cell line had been incubated with either U937 CM or LPS-activated U937 CM, the sphere formation assay showed a significant increase in HCP-1 sphere-forming capability. In particular, LPS-activated U937 CM increased the number of spheres by more than four-fold compared to control CM (Fig 4A, P < 0.001). In addition, LPS-activated U937 CM treatment enriched the Aldefluor-positive cell population of HCP-1 by two-fold compared to control CM (Fig 4B, P < 0.01). As expected, a Western blot analysis of the different CM showed that SHH was secreted at a higher rate by LPS-activated U937 cells compared to HCP-1 cells and U937 non-treated cells (Fig 4C). We also confirmed these results using human monocytes isolated from normal donor buffy coat. The results corroborated our previous findings: 1) the sphere formation assay showed a significantly increase in HCP-1 sphere-forming ability (6-fold) (Fig 4D; P < 0.01); 2) LPS-activated macrophage CM treatment enriched the Aldefluor-positive HCP-1 cell population by more than 3-fold compared to control CM (Fig 4E; P < 0.001); 3) Western blot analysis of the different CMs showed that SHH was secreted at a higher levels by LPS- activated macrophage compared to HCP-1 cells and untreated monocytes (Fig 4F).

## Discussion

In the growing fields of immunotherapy and the tumor microenvironment, conflicting studies about the role of macrophages in tumor progression warrant continued investigation, especially in CRC. In this study, we examined the effect of classically LPS-activated macrophage CM on CRC cell lines, our results showed that LPS-activated macrophage CM mediates the CSC phenotype through activation of the SHH-Gli signaling pathway in CRC cells. Furthermore, we demonstrated that LPS-activated macrophage CM enhanced chemoresistance. Because macrophages display a broad functional spectrum and can change function depending on the microenvironment, their effects on tumor progression are difficult to predict. Such conflicts could be partly explained by the way macrophages are polarized by the tumor microenvironment [49]. Although TAMs were initially believed to be involved in mediating the pro-tumor activities of the M2 phenotype, it is now understood that they are probably composed of several distinct populations, with overlapping M1-like and M2-like features; these phenotypes may be dependent upon a variety of factors, including location in the microenvironment, stage of the tumor, and type of cancer [31, 50, 51]. Thus, the restrictive classification of TAMs simply as M1 or M2 does not accurately reflect their biological state *in vivo*. Our in vitro studies also demonstrated that LPS-activated macrophages were actually a mixture of both phenotypes (S1 Fig). This supports the hypothesis that macrophage activation leads to a mixed or double phenotype of M1 and M2 macrophages with populations likely ranging from one extreme to the other and also various phenotypes in between.

A growing body of evidence suggests that TAMs are critical for the self-renewal and maintenance of CSCs in established tumors [29–31]. Hedgehog (HH) is one of the major mediators involved in self-renewal and maintenance of CSCs [52]. The importance of this pathway has been demonstrated in various cancers including breast, lung, pancreas, colon and hepatic cancers [52]. Briefly, the HH pathway is activated by a family of ligands, three of which have been identified in humans: Sonic hedgehog (SHH), Indian hedgehog (IHH) and Desert hedgehog (DHH). Of these, SHH has been extensively studied [53]. Patched-1 (PTCH1), the receptor of SHH, inhibits Smoothened (SMO), a downstream protein in the HH pathway. However, upon SHH binding the inhibition of SHO by PTCH1 is relieved. These events lead to subsequent activation of the GLI transcription factors: the activators Gli1 and Gli2, and the repressor Gli3 [53]. In this study, we showed for the first time, that even classically activated macrophages are able to enrich the CSC population of CRC cell lines and that this enrichment is mediated by the SHH protein that is present in the macrophage CM. A previous study indicated that TAMs express milk-fat globule-epidermal growth factor-V111 (MFG-E8) that leads to enhanced pSTAT3 and SMO in CRC CSCs [35]. Increase in SMO in CSCs were linked to increased activity of the SHH pathway, an important regulator of stemness. Our observation that the LPS-activated macrophage CM itself contains high amounts of SHH and that treating CRC cells with this CM induces significant increases in GLI activity and increase in stem cell population, suggests a novel and more direct mechanism of activation of the SHH pathway. However, we have not been able to determine whether this increase in the relative CSC population was due to selection of CSCs or by direct stimulation of the CSC phenotype of CRC.

In summary, our findings emphasize the complexity of macrophage biology in the tumor microenvironment. Our results underline, 1) the ability of classically activated macrophages to stimulate the CSC phenotype in CRC cells, and, 2) LPS-activated macrophage CM promotes chemoresistance of CRC cells. Since macrophages are able to stimulate the CSC phenotype and promote chemoresistance, our results add to the consensus that macrophages are of critical importance to tumors response to treatment and that due to their plasticity can influence tumor growth and possibly, response to treatment. The multifactorial roles of macrophages are key in tumor-promoting or tumor-suppressing processes and should be considered overall in the design of anti-tumor therapeutics.

## Supporting information

S1 Fig Reverse transcription polymerase chain reaction (RT-PCR). Characterization of the LPS-activated macrophage population through the detection of canonical M1 and M2 macrophage polarization markers *in vitro*.
(TIF)

S2 Fig Macrophage CM activates SHH signaling in CRC cells. Promoter activity of Hes-1, Gli, and TCF in HCP-1 cells after treatment with control CM or macrophage CM.
(TIF)

S1 Table Primers used for the RT-PCR. f: forward; r: reverse.
(TIF)
