# Supplementary figures and images for: Macrophage conditioned medium promotes colorectal cancer stem cell phenotype via the hedgehog signaling pathway
Source: PLoS One. 2018 Jan 2;13(1):e0190070. doi: 10.1371/journal.pone.0190070 (PMC5749743; doi:10.1371/journal.pone.0190070)

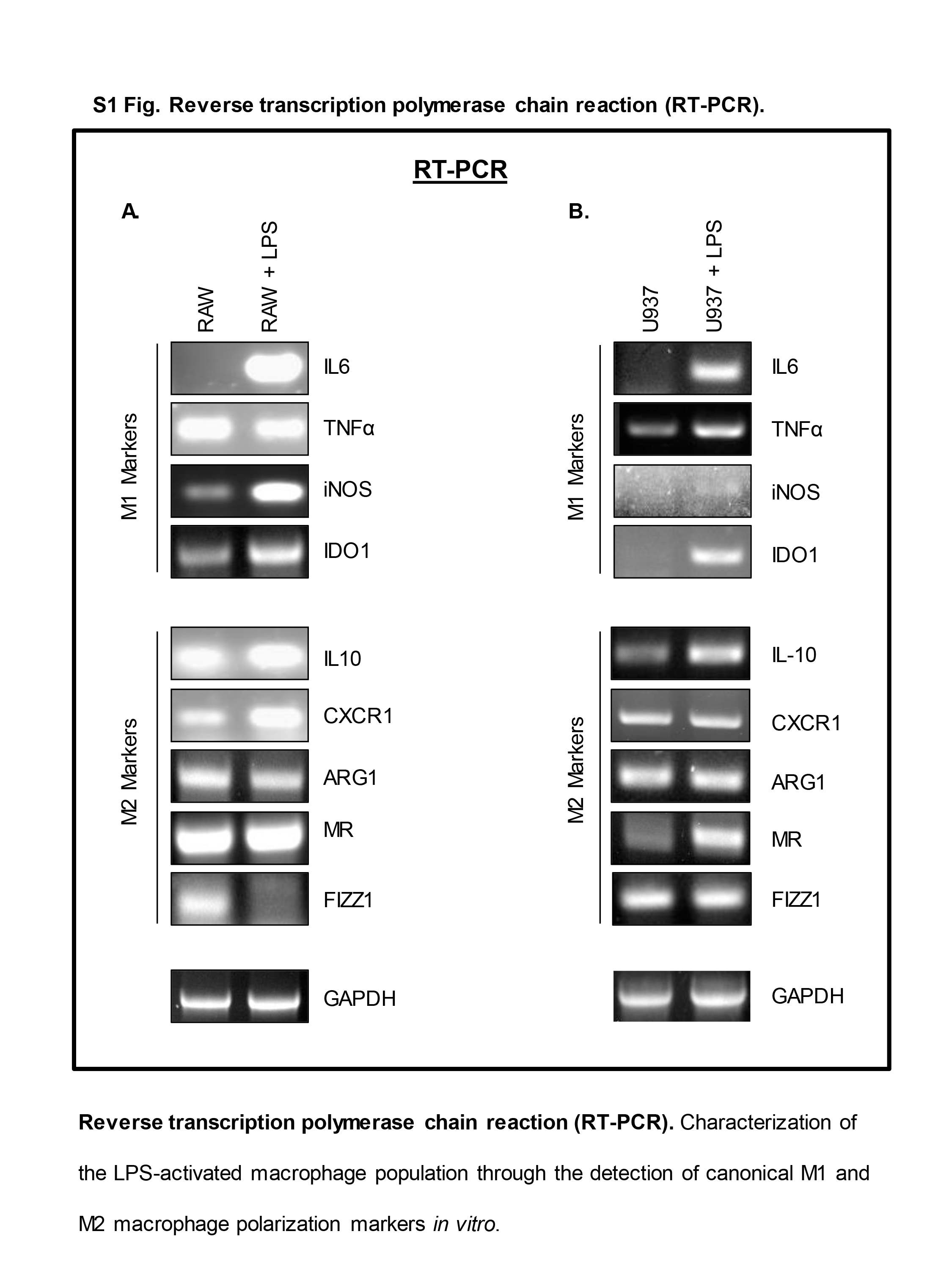

Supplement: S1 Fig — Characterization of the LPS-activated macrophage population through the detection of canonical M1 and M2 macrophage polarization markers in vitro. (TIF) [file pone.0190070.s001.tif]

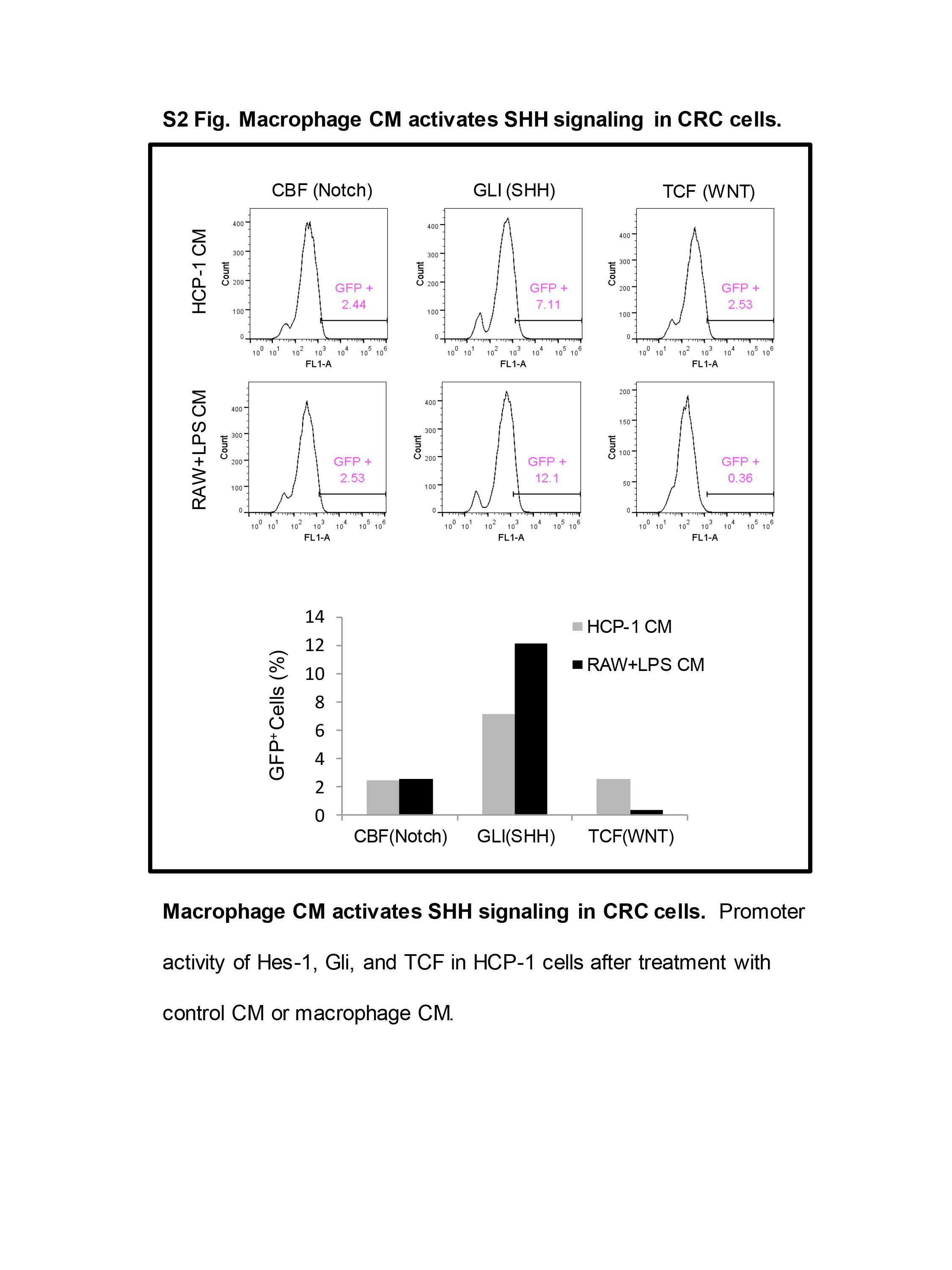

Supplement: S2 Fig — Promoter activity of Hes-1, Gli, and TCF in HCP-1 cells after treatment with control CM or macrophage CM. (TIF) [file pone.0190070.s002.tif]

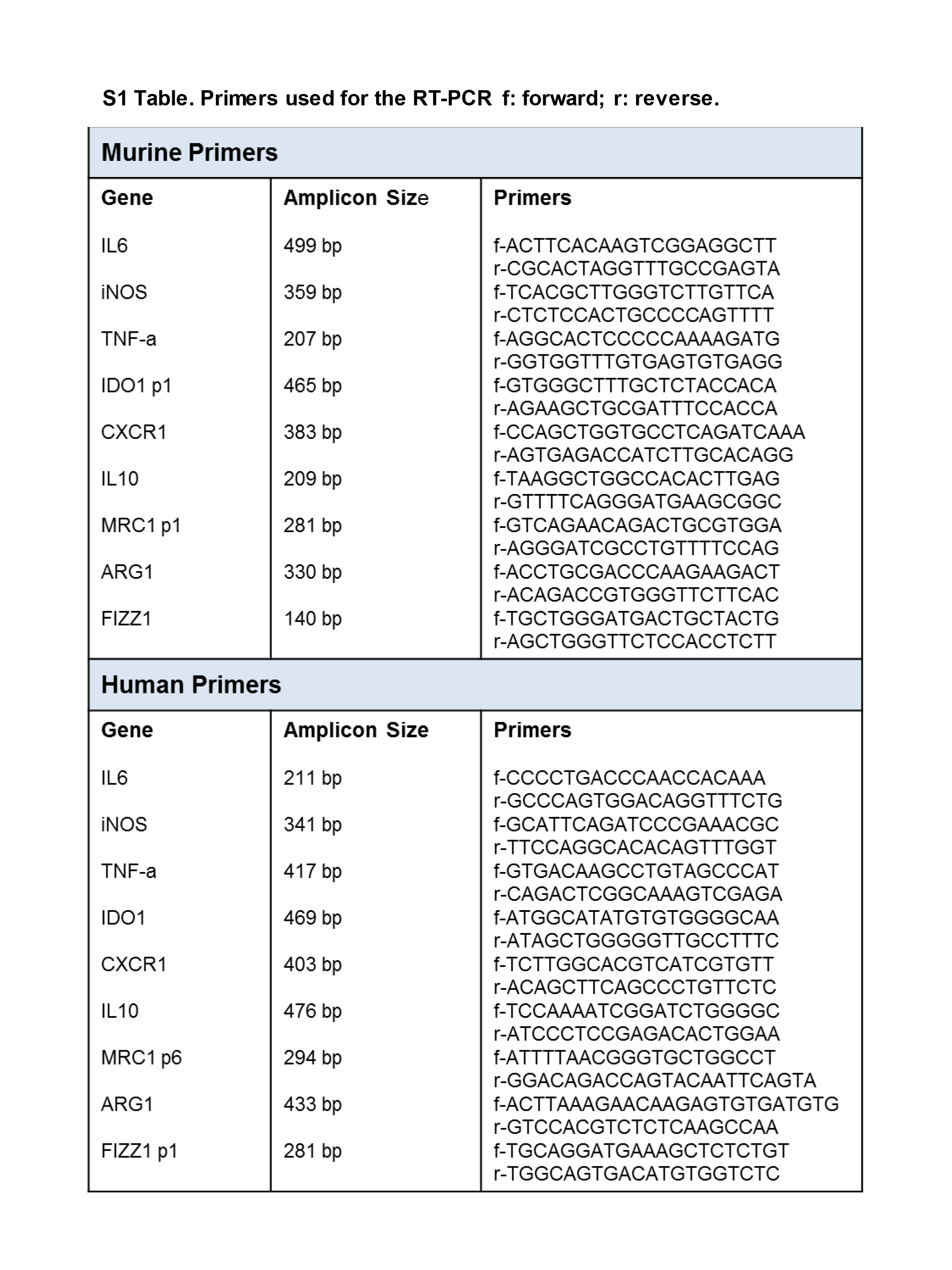

Supplement: S1 Table — f: forward; r: reverse. (TIF) [file pone.0190070.s003.tif]
